# Identification of key genes and miRNAs related to polycystic ovary syndrome by comprehensive analysis of microarray

**DOI:** 10.1186/s12920-022-01384-9

**Published:** 2022-12-21

**Authors:** Ziqian Sun, Yang Wang, Tianshu Wei, Li Liu

**Affiliations:** 1 Department of Gynecology and Obstetrics, China-Japan Union Hospital of Jilin University, Changchun, 130033 Jilin Province China; 2 Department of Dermatology, Bayi Hospital of Changchun, Changchun, 130021 Jilin Province China; 3 Department of Gynecology and Obstetrics, Jilin City Center Hospital, Jilin City, 132011 Jilin Province China; 4 Reproductive Medical Center, China-Japan Union Hospital of Jilin University, No. 126, Xiantai Road, Changchun, 130031 China

**Keywords:** Polycystic ovary syndrome, Differentially expressed genes, Differentially expressed miRNAs, PPI network analysis

## Abstract

**Background:**

We aimed to explore mechanisms of development and progression of polycystic ovary syndrome (PCOS).

**Methods:**

The microRNA expression microarray GSE37914 and gene expression profiles GSE43264 and GSE98421 were downloaded from the Gene Expression Omnibus database. The differentially expressed miRNAs (DEmiRNAs) and genes (DEGs) were screened using Limma package. Then, the DEGs and DEmiRNAs were combined to use for the subsequent analysis, including the functional enrichment analysis, protein–protein interaction (PPI) network and module analysis, drug–gene interaction network analysis, and DEmiRNAs–DEGs interactive network construction.

**Results:**

A total of 26 DEmiRNAs and 80 DEGs were screened. The PPI network contained 68 nodes and 259 interactions. A significant clustering module with 8 nodes and 25 interactions was obtained. Three PCOS-related overlapping pathways were obtained based on PPI-degree top10 and module genes, including prion diseases, Staphylococcus aureus infection, and Chagas disease (American trypanosomiasis). A total of 44 drug–gene interaction pairs were obtained, which included 2 up-regulated genes (LDLR and VCAM1), 4 down-regulated genes (C1QA, C1QB, IL6 and ACAN) and 26 small molecules drugs. A total of 52 nodes and 57 interactions were obtained in the DEmiRNA–DEGs regulatory network, LDLR was regulated by miR-152-3p, miR-1207-5p, miR-378a-5p and miR-150-5p.

**Conclusions:**

Our research has identified several key genes and pathways related to PCOS. These results can improve our understanding of PCOS and provide new basis for drug target research.

## Background

Polycystic ovary syndrome (PCOS) is a common endocrinological disorder disease in women, which affected 4–10% women worldwide, and increased the risk of reproductive abnormalities [1, 2]. The syndrome is related to numerous morbidities, such as obstetrical complications, infertility, cardiovascular disease, and type 2 diabetes mellitus [3, 4]. Many compensatory hyperinsulinemia can lead to hyperandrogenemia by stimulating ovarian androgen secretion and inhibiting the production of hepatic sex hormone binding globulin [5]. Genetic and environmental factors also play an important role in the progression of PCOS [6, 7]. Adipose tissue dysfunction is one of the causes of insulin resistance in PCOS. Obesity dependent insulin resistance is associated with PCOS. The prevalence of obesity in women with PCOS is higher than that in women of the same age without this syndrome, but the risk of PCOS only slightly increases with obesity [8]. In addition, visceral adiposity alone could not account for differences in insulin sensitivity between women with PCOS and those without the syndrome, though the problem is controversial [9]. The potential mechanism of insulin resistance in polycystic ovary syndrome is still unclear. Insulin resistance in patients with PCOS may be due to impaired local adipose tissue storage ability resulting in dyslipidemia, which leads to improved dietary caloric intake [10, 11]. The parasecretory imbalance of cytokines secreted by macrophages in patients with polycystic ovary syndrome is conducive to the occurrence of insulin resistance [12]. Understanding the underlying mechanisms of adipose tissue dysfunction and insulin resistance in PCOS may eventually find new therapeutic targets for PCOS.

MicroRNAs (miRNAs) are a class of single-stranded, small noncoding RNAs of approximately 22 nucleotides in length that are involved in the processes of biological growth, development, differentiation, and disease. Most human protein-coding genes are regulated by miRNAs, which are involved in the occurrence, development and almost all life activities of diseases. In recent years, studies have shown that miRNAs are closely related to ovarian physiology and pathology, and the relationship between miRNAs and PCOS was studied by screening the differential expression of miRNAs in ovarian tissues [13]. However, miRNAs as biomarkers for PCOS remain to be further investigated.

In this study, the differentially expressed miRNAs (DEmiRNAs) from GSE37914 and the differentially expressed genes (DEGs) from GSE43264 and GSE98421 were screened first. Subsequently, integrated bioinformatics analyses, including the Kyoto Encyclopedia of Genes and Genomes (KEGG) pathway enrichment analysis, the network analysis of protein–protein interaction (PPI), drug–gene interaction network and miRNAs–target genes network were performed. This research may help to explore the mechanism of PCOS and screen candidate biomarkers and treatment targets.

## Methods

### Microarray data and data preprocessing

PCOS, which is diagnosed as a diagnosis of exclusion. The most commonly used diagnostic criteria are the Rotterdam criteria proposed in 2003, which are as follows: 1. Rare ovulation or anovulation; 2. Clinical manifestations of hyperandrogenism and hyperandrogenism; 3. Changes in polycystic ovaries, as indicated by ultrasound One or both ovaries, more than 12 follicles with a diameter of 2–9 mm, and ovarian volume greater than 10 mL; 4. Two of the three items are met, and other causes of hyperandrogenism, congenital adrenal hyperplasia and Cushing's syndrome are excluded, androgen-secreting tumors, you can diagnose PCOS. The miRNA expression microarray data set GSE37914 and gene expression profiles GSE43264 and GPL15362 were obtained from Gene Expression Omnibus (GEO). The GSE37914 contained 6 subcutaneous adipose tissue samples, 3 patients with the lean PCOS and 3 matched control women, and the platform used was GPL8786[miRNA-1] Affymetrix Multispecies miRNA-1 Array. GSE43264 contained 15 subcutaneous adipose tissue samples, 8 women with PCOS and 7 matched healthy controls, and the platform used was GPL15362 NuGO array (human) NuGO_Hs1a520180 [CDF: Hs_ENTREZG_14]. The GSE98421 GSE98421 contained 8 subcutaneous adipose tissue samples from PCOS (n = 4) and NL (n = 4), and the platform used was GPL570 [HG-U133_Plus_2] Affymetrix Human Genome U133 Plus 2.0 Array. The three dataset were preprocessed, which included background correction, quantile normalization and probe summarization, using Oligo package (version 1.34.0) in R language [14] and limma (Version 3.10.3) package [15].

### Identification of DEGs and pathway analysis

The three normalized data were calculated by limma (Version 3.10.3) package. Genes with P < 0.05 and |logFC (fold change)|> 1 were regarded as DEGs between the PCOS group and the normal group, and miRNAs with P < 0.05 and |logFC (fold change)|> 1 were considered as DEmiRNAs. The heatmap of DEGs and DEmiRNAs were carried out using pheatmap package (version 1.0.10) in R language. The DEGs of GSE43264 and GSE98421 were combined to use for the subsequent analysis.

Pathway enrichment analysis [16] of DEGs and the subsequent PPI degree top10 was performed using the ClusterProfiler package (Version 2.4.3) in R [17], and the significant enrichment pathways with the cutoff of P < 0.05 and the count > 2. In addition, the pathways correlated with PCOS and Obesity were searched using the CTD (Comparative Toxicology Database) [18], and the search pages of PCOS and Obesity were http://ctdbase.org/detail.go?type=disease&acc=MESH%3aD011085&view=pathway and http://ctdbase.org/detail.go?type=disease&acc=MESH%3aD009765&view=pathway, respectively.

### Analysis of PPI network

String database (Version: 11.0) [19]was used to predict and analyze the interaction between proteins encoded by genes, and the DEGs in the PPI network was established through the String and cyberscape software. In order to obtain as many relationship pairs as possible, the parameter PPI score is set as 0.15 (representing low confidence index). By analyzing the topological properties of network nodes, the gene with higher degree value is obtained, namely hub gene. The plug-in Mcode [20] (Version 1.4.2) of cytoscape was used to analyzed the most remarkable clustering module in PPI network and the threshold score ≥ 5 was selected in this study.

### Drug–gene interaction network

The Drug Gene Interaction Database (DGIdb, http://www.dgidb.org/), is a web resources that could be applied for searching candidate drugs or genes against the known and potentially druggable genome. We used DGIDB 2.0 [21] to predict the drug–gene relationship pairs of the top ten genes from PPI network, and the network of drug–gene relationship pairs was visualized using Cytoscape (version 3.2.0, http://www.cytoscape.org/).

### DEmiRNAs–DEGs interaction analysis

All up-regulated miRNAs and top10 down-miRNAs with larger foldchange were predicted using miRWalk 2.0 (http://zmf.umm.uni-heidelberg.de/apps/zmf/mirwalk2/) [22], including miRWalk, miRanda, miRDB, miRMap, mirmap, Pictar2, RNA22, and Targetscan, and the miRNA-target gene pairs at least 5 databases were screened and used for the subsequent analysis. The miRNA-target gene pairs and the DEGs were combined, then Cytoscape was used to construct DEmiRNAs–DEGs interaction network.

### Statistical analysis

Data were analyzed by SPSS 21.0 (SPSS Inc., Chicago, IL, USA) and expressed as mean ± standard division. The continuous variables were compared using unpaired Student’s t tests, and chi-square tests were used for nominal parameters. *P* value < 0.05 was considered statistically significant.

## Results

### Identification of DEGs and DEmiRNAs

**Fig. 1:**
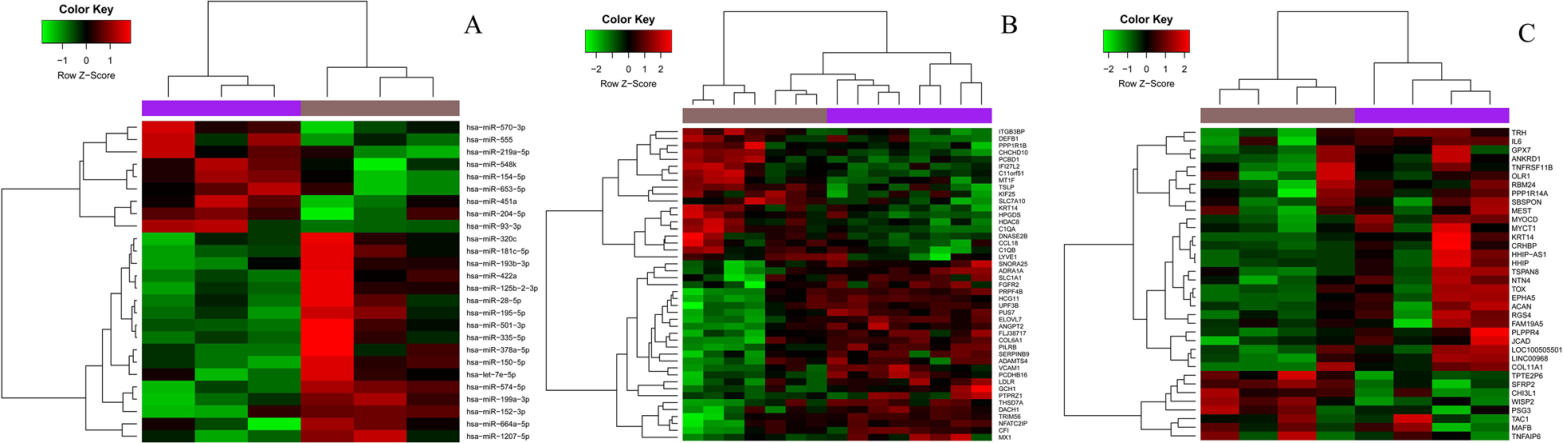
The heatmap of differentially expressed genes and miRNAs (DEGs, DEmiRNAs) between the PCOS group and the normal group were analyzed. The heatmap of DEmiRNAs in GSE37914 was shown in **A**, and the heatmap of DEGs in GSE43264 and GSE98421 was shown in **B** and **C**, respectively. Brown represented the control group and purple represented the disease group

A total of 26 DEmiRNAs in the GSE37914 was identified, including 9 up-regulated and 17 down-regulated DEmiRNAs, and the distribution of DEmiRNAs was shown in Fig. 1A. A total of 45 and 26 DEGs were obtained in the GSE43264 and GSE98421 datasets, respectively. The heatmap of DEGs was shown in Fig. 1B and C. We merged the DEGs of the two datasets GSE43264 and GSE98421, and obtained a total of 80 DEGs, including 34 up-regulated and 46 down-regulated DEGs (KRT14 is the only overlapped down-regulated gene). The 80 DEGs were used for the subsequent analysis.

### KEGG pathway analysis of DEGs

As shown in Table 1, the pathways of 34 up-regulated DEGs were involved in protein digestion and absorption (*P* = 1.58e^−02^), PI3K-Akt signaling pathway (*P* = 3.87e^−02^), and hepatitis C (*P* = 4.35e^−02^); and the The pathways of 46 down-regulated DEGs were involved in prion diseases (*P* = 1.18e^−04^), staphylococcus aureus infection (*P* = 8.53e^−04^), pertussis (*P* = 1.18e^−03^), chagas disease (American trypanosomiasis) (*P* = 2.83e^−03^), cytokine–cytokine receptor interaction (*P* = 8.29e^−03^), glutathione metabolism (*P* = 1.06e^−02^), arachidonic acid metabolism (*P* = 1.32e^−02^), and complement and coagulation cascades (*P* = 2.03e^−02^). The 11 pathways are listed in CTD as being related to obesity, and eight pathways are also related to PCOS, including, including PI3K-Akt signaling pathway, hepatitis C, prion diseases, staphylococcus aureus infection, chagas disease (American trypanosomiasis), cytokine–cytokine receptor interaction, glutathione metabolism, and arachidonic acid metabolism.

**Table 1:** The enrichment result of KEGG pathway of the differentially expressed genes

	ID	Description	Count	*P* value	Genes
Up	hsa04974	Protein digestion and absorption	2	1.58E-02	COL6A1/SLC1A1
	hsa04151	PI3K-Akt signaling pathway	3	3.87E-02	COL6A1/ANGPT2/FGFR2
	hsa05160	Hepatitis C	2	4.35E-02	LDLR/MX1
Down	hsa05020	Prion diseases	3	1.18E-04	C1QB/C1QA/IL6
	hsa05150	Staphylococcus aureus infection	3	8.53E-04	C1QB/C1QA/DEFB1
	hsa05133	Pertussis	3	1.18E-03	C1QB/C1QA/IL6
	hsa05142	Chagas disease (American trypanosomiasis)	3	2.83E-03	C1QB/C1QA/IL6
	hsa04060	Cytokine–cytokine receptor interaction	4	8.29E-03	CCL18/TSLP/IL6/TNFRSF11B
	hsa00480	Glutathione metabolism	2	1.06E-02	HPGDS/GPX7
	hsa00590	Arachidonic acid metabolism	2	1.32E-02	HPGDS/GPX7
	hsa04610	Complement and coagulation cascades	2	2.03E-02	C1QB/C1QA

### Analysis of PPI network

**Fig. 2:**
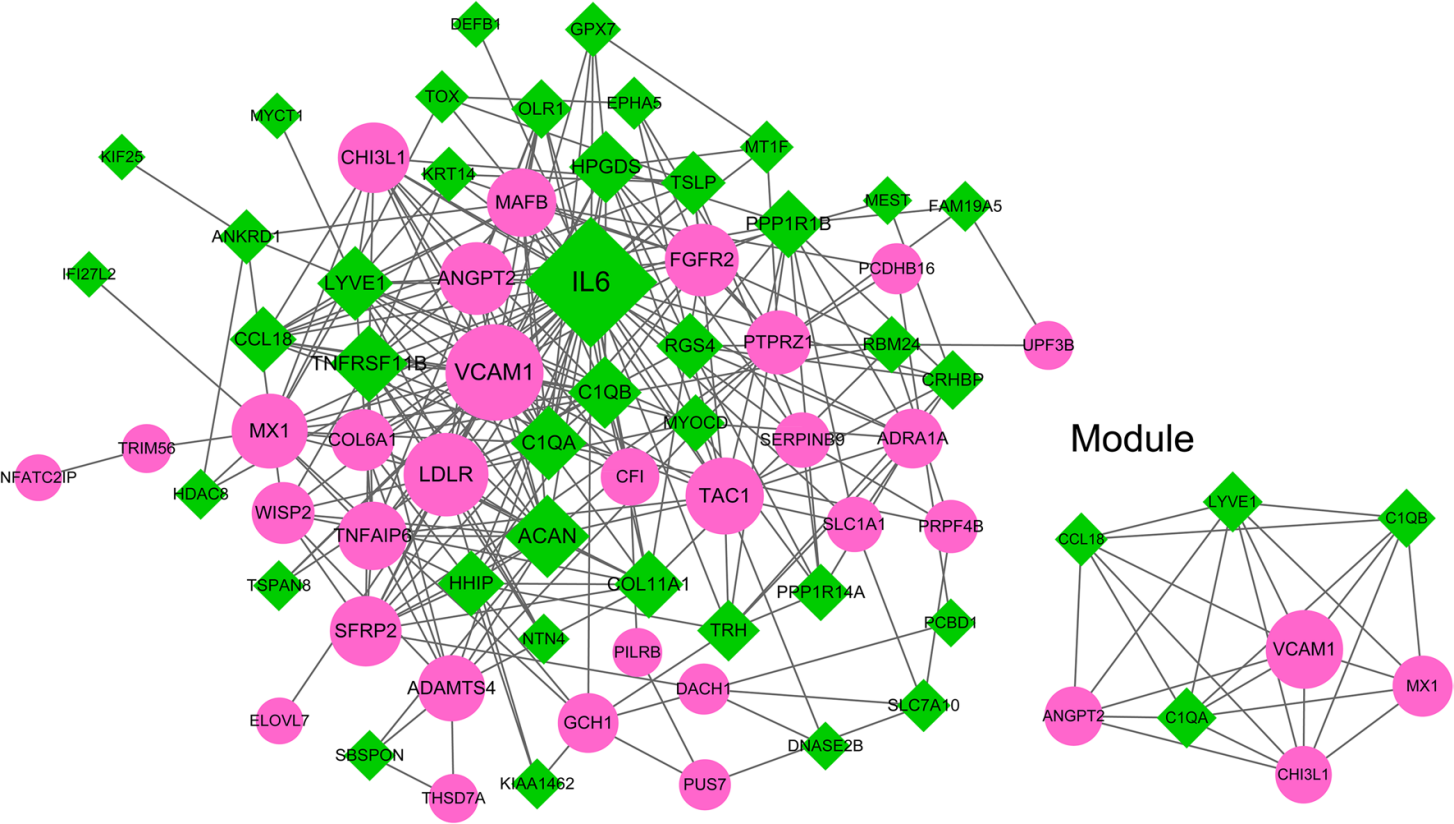
The PPI network of differentially expressed genes. Red circles represented up-regulated genes and green rhombus represented down-regulated genes. The node size represented degree values, and the larger node size implied the larger degree value

We constructed PPI network for DEGs, and the result was shown in Fig. 2. The network included 68 nodes and 259 pairs of interaction relations. According to the module threshold, the only one significant clustering module (score = 7.143) was obtained, which contains 8 nodes and 25 interaction pairs. The top 10 degree genes were IL6, VCAM1, ACAN, LDLR, TAC1, TNFRSF11B, C1QA, MX1, LYVE1, and C1QB, and the module genes were VCAM1, C1QA, LYVE1, MX1, ANGPT2, C1QB, CHI3L1 and CCL18 (Table 2).

**Table 2:** The top10 degree genes and module genes in PPI network

PPI-degree top10			Module		
Nodes	Description	Degree	Nodes	Description	Degree
IL6	DOWN	40	VCAM1	UP	24
VCAM1	UP	24	C1QA	DOWN	14
ACAN	DOWN	18	LYVE1	DOWN	14
LDLR	UP	18	MX1	UP	14
TAC1	UP	15	ANGPT2	UP	13
TNFRSF11B	DOWN	15	C1QB	DOWN	13
C1QA	DOWN	14	CHI3L1	UP	12
MX1	UP	14	CCL18	DOWN	10
LYVE1	DOWN	14			
C1QB	DOWN	13			

KEGG pathway analysis was performed to further explore the functions of the PPI-degree top 10 hub genes and module genes respectively. Totally, 14 pathways were obtained by PPI-degree TOP10 genes and 6 by module genes (Table 3). The search results in CTD database implied that the all pathways were correlated with Obesity, and the 8 of 20 pathways were related to PCOS, including prion diseases, chagas disease (American trypanosomiasis), staphylococcus aureus infection, AGE-RAGE signaling pathway in diabetic complications, TNF signaling pathway, measles, hepatitis C, and cytokine–cytokine receptor interaction. Among the 8 PCOS-related pathways, prion diseases, Staphylococcus aureus infection, and Chagas disease (American trypanosomiasis) were overlapping pathways in the PPI-degree TOP10 genes and module genes.

**Table 3:** The enrichment result of KEGG pathway of the top10 degree genes and module genes in PPI network

	ID	Description	Count	*P* value	Genes
PPI-degree top 10	hsa05020	Prion diseases	3	4.49E-06	IL6/C1QA/C1QB
	hsa05133	Pertussis	3	4.73E-05	IL6/C1QA/C1QB
	hsa05142	Chagas disease (American trypanosomiasis)	3	1.17E-04	IL6/C1QA/C1QB
	hsa05143	African trypanosomiasis	2	5.96E-04	IL6/VCAM1
	hsa05144	Malaria	2	1.05E-03	IL6/VCAM1
	hsa05150	Staphylococcus aureus infection	2	2.01E-03	C1QA/C1QB
	hsa04610	Complement and coagulation cascades	2	2.70E-03	C1QA/C1QB
	hsa04933	AGE-RAGE signaling pathway in diabetic complications	2	4.29E-03	IL6/VCAM1
	hsa04668	TNF signaling pathway	2	5.17E-03	IL6/VCAM1
	hsa05322	Systemic lupus erythematosus	2	7.47E-03	C1QA/C1QB
	hsa05162	Measles	2	8.03E-03	IL6/MX1
	hsa05160	Hepatitis C	2	1.00E-02	LDLR/MX1
	hsa05164	Influenza A	2	1.16E-02	IL6/MX1
	hsa04060	Cytokine–cytokine receptor interaction	2	3.38E-02	IL6/TNFRSF11B
Module	hsa05020	Prion diseases	2	2.87E-04	C1QA/C1QB
	hsa05150	Staphylococcus aureus infection	2	1.09E-03	C1QA/C1QB
	hsa05133	Pertussis	2	1.36E-03	C1QA/C1QB
	hsa04610	Complement and coagulation cascades	2	1.46E-03	C1QA/C1QB
	hsa05142	Chagas disease (American trypanosomiasis)	2	2.48E-03	C1QA/C1QB
	hsa05322	Systemic lupus erythematosus	2	4.09E-03	C1QA/C1QB

### Drug–gene interaction analysis

**Fig. 3:**
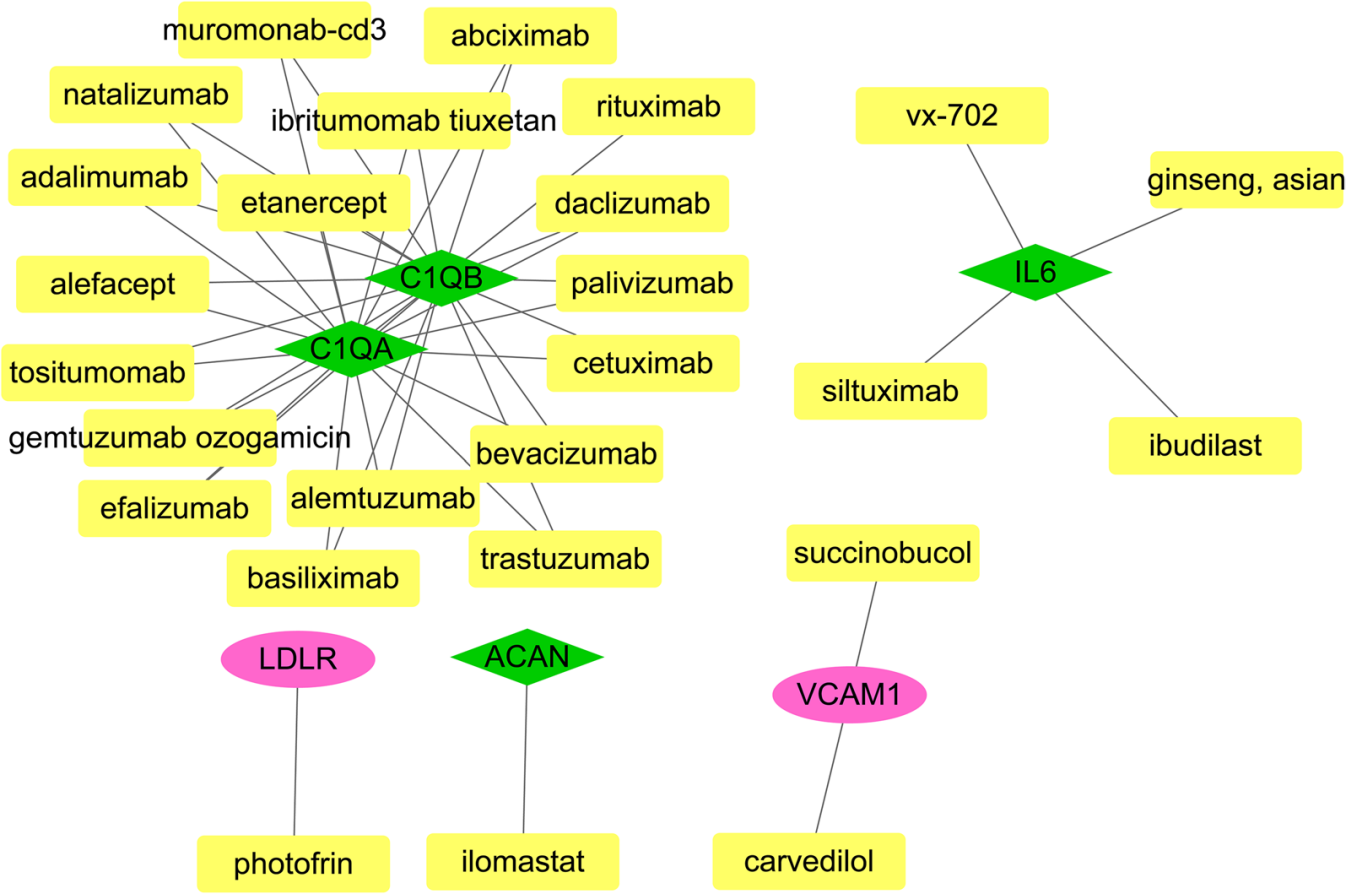
The drug–gene network network of differentially expressed genes and drugs. Red circle represented up-regulated genes, green rhombus represented down-regulated genes, and yellow rectangle represented drug

Based on the DGIdb prediction results of PPI-degree top 10 and module genes, a total of 44 drug–gene interaction pairs were obtained. In addition, 26 small molecules drugs were predicted to be implicated with 2 up-regulated genes (LDLR and VCAM1) and 4 down-regulated genes (C1QA, C1QB, IL6 and ACAN) using DGIdb (Fig. 3).

### miRNAs–DEGs interaction analysis

**Fig. 4:**
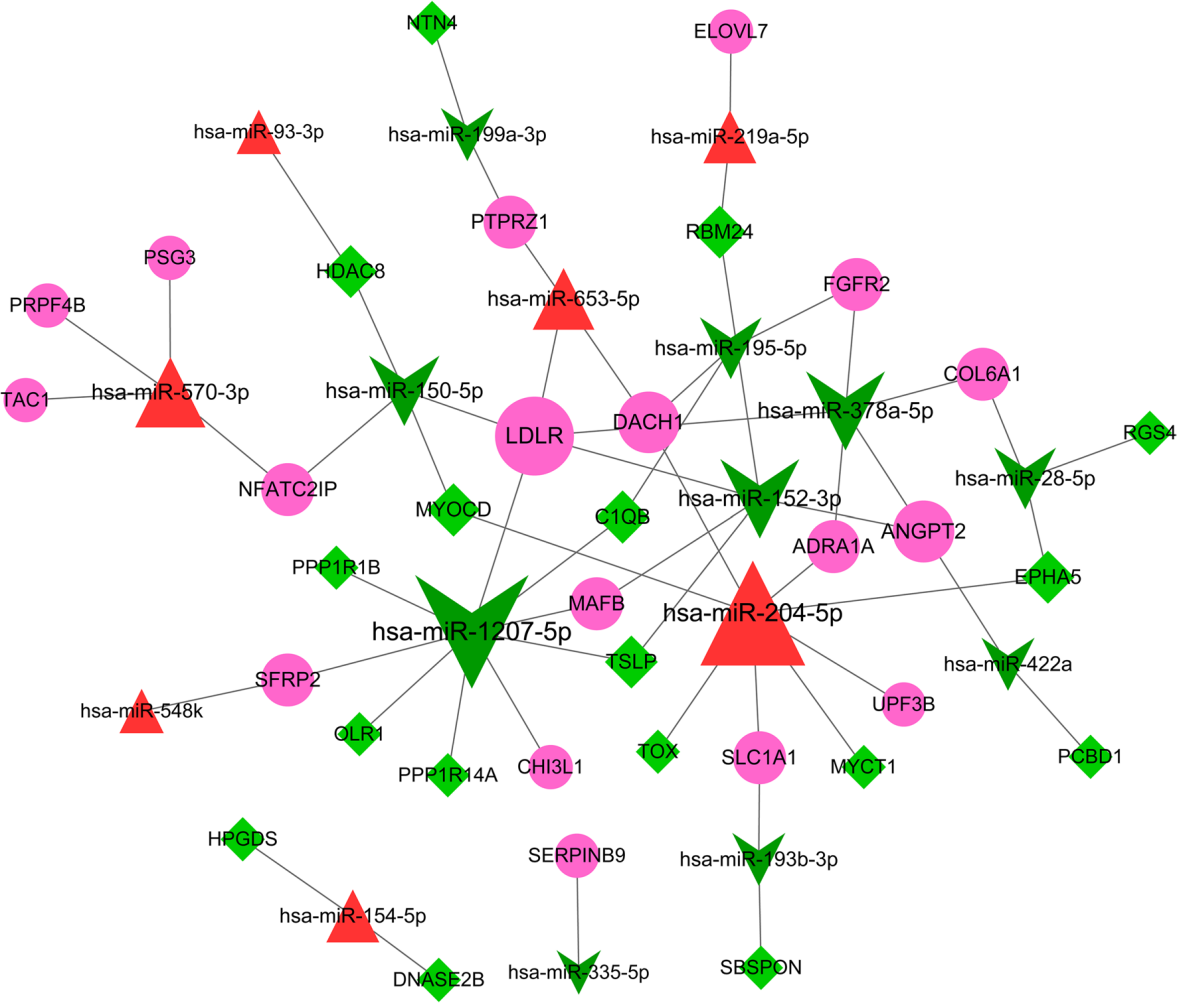
The differentially expressed miRNAs and target genes regulatory network. Red triangle indicated up-regulated miRNA, green arrow indicated down-regulated miRNAs, red circle indicated up-regulated genes, and green rhombus indicated down-regulated genes

In total, 52 nodes and 57 DEmiRNAs-DEGs relationship pairs were obtained, including 7 up-regulated miRNAs, 10 down-regulated miRNAs, 18 up-regulated genes and 17 down-regulated genes (Fig. 4). From the regulatory network, 52 nodes and 57 pairs of interactions were observed, The DEmiRNA and DEGs with degree value TOP10 were shown in Table 4, where LDLR interacted with miR-152-3p, miR-1207-5p, miR-378a-5p and miR-150-5p.

**Table 4:** The results of DEmiRNA and DEGs of degree top10

Nodes	Description	Degree
hsa-miR-1207-5p	DOWN-miRNA	9
hsa-miR-204-5p	UP-miRNA	8
hsa-miR-152-3p	DOWN-miRNA	5
hsa-miR-378a-5p	DOWN-miRNA	5
LDLR	UP-gene	5
hsa-miR-150-5p	DOWN-miRNA	4
hsa-miR-570-3p	UP-miRNA	4
hsa-miR-28-5p	DOWN-miRNA	3
hsa-miR-195-5p	DOWN-miRNA	3
DACH1	UP-gene	3

## Discussion

PCOS is a common endocrine disorder in women with an increasing risk of reproductive abnormalities. In the current study, a total of 26 DEmiRNAs were identified in the GSE37914, and 80 DEGs were selected from GSE43264 and GSE98421. Prion diseases, Staphylococcus aureus infection, and Chagas disease (American trypanosomiasis) were overlapping pathways in the PPI-degree TOP10 genes and module genes, the enrichment of these pathways may be due to the immune process involved. 6 hub genes were identified by drug–gene interaction analysis, which were *LDLR*, *VCAM1, C1QA*, *C1QB*, *IL6* and *ACAN*. In the regulatory network of DEmiRNAs and DEGs, *LDLR* was regulated by miR-1207-5p, miR-152-3p, miR-378a-5p and miR-150-5p.

PCOS is characterized by obesity, hypertension, insulin resistance, diabetes mellitus and other metabolic diseases. The serum IL-6 level and the secretion of LPS activated monocytes are increased in insulin resistant PCOS patients [23]. In addition, the increase of IL-6 level in obese women with PCOS, which is not associated with obesity, may be related to insulin resistance [24]. VCAM-1 aggravates PCOS symptoms by improving leukocyte recruitment to the ovary and sustaining local inflammation [25]. Low density lipoprotein receptor (LDLR) has been proved to play an important role in lipoprotein metabolism. The number of follicles in LDLR − / − mice was less, the follicular atresia was more, the estrogen level was lower, and the estrus time was significantly shorter than that of the control group [26]. The previously reports suggested that LDLR played a central role in uptake and clearance of LDL cholesterol. Expression levels of LDLR mRNAs were markedly expressed in the PCOS NAFLD group when contrasted with the non-PCOS NAFLD group [27]. Complement component 1q (C1q) is the starting protein of the classical complement pathway. Complement C1q A chain (*C1QA*) and complement C1q B chain (*C1QB*) encode the a chain and b chain polypeptides of serum complement subcomponent C1q, respectively. The results of Katherine's research showed that the classical complement pathway was activated in the kidneys of diabetic rats, and the *C1QA* and *C1QB* were significantly high expression [28]. *C1QA* and *C1QB* interact with a variety of small drug molecules and may be potential targets for drug development.

In addition, the network analysis of miRNAs-target genes network to explore mechanisms of development and progression of PCOS. Plasma levels of miR-152-3p were related to diabetic nephropathy [29]. The reports implied that hsa-miR-1207-5p had a important role in many diseases, such as monogenic renal disorder [30] and serous ovarian carcinoma [31]. Some studies have shown that miR-378a is an important regulator of energy and glucose homeostasis and a potential target for improving metabolic disorders [32]. The results of Kang et al. indicate that MIR-150 may be a biomarker and new therapeutic target for obesity and insulin resistance [33].

## Conclusions

In conclusion, our research provides a comprehensive bioinformatics analysis of PCOS, LDLR, VCAM1, C1QA, C1QB, IL6 and ACAN6 may be potential targets for drug development of PCOS. LDLR, miR-152-3p, miR-1207-5p, miR-378a-5p and miR-150-5p might involve in the pathogenesis of PCOS.

## Data Availability

The datasets generated and analysed during the current study are available in the National Center for Biotechnology Information Gene Expression Omnibus (GEO) repository. GSE37914: https://www.ncbi.nlm.nih.gov/geo/query/acc.cgi?acc=GSE37914. GSE43264: https://www.ncbi.nlm.nih.gov/geo/query/acc.cgi?acc=GSE43264. GSE98421: https://www.ncbi.nlm.nih.gov/geo/query/acc.cgi?acc=GSE98421.

## Abbreviations

LDL: Low-density lipoprotein
LDLR: Low-density lipoprotein receptor
PCOS: Polycystic ovary syndrome
VCAM1: Vascular cell adhesion molecule 1
NAFLD: Non-alcoholic fatty liver disease
DGIdb: Drug Gene Interaction Database
KEGG: Kyoto Encyclopedia of Genes and Genomes
PPI: Protein–protein interaction
